# Thyroid Storm

**DOI:** 10.21980/J8RW71

**Published:** 2020-10-15

**Authors:** Kathryn Ritter, Carmen Wolfe

**Affiliations:** * Vanderbilt University Medical Center, Department of Emergency Medicine, Nashville, TN

## Abstract

**Audience:**

This is a practice oral boards case which may be given to emergency medicine (EM) residents at all levels of training and recent EM graduates.

**Introduction/Background:**

Thyroid storm is an acute, life-threatening endocrine emergency. It occurs when there is excess circulating thyroid hormone in the bloodstream. It may be precipitated by infection, surgery, pregnancy, trauma, thyroid medication changes, or iodinated contrast exposure. This condition must be quickly identified and treated by EM physicians in order to prevent morbidity and mortality. IThe mortality rate is between 10 and 30%^1^ Understanding and treating thyroid storm is included in the 2019 Model of Clinical Practice of Emergency Medicine.^2^

**Educational Objectives:**

At the end of this practice oral boards case, the learner will: 1) assess a patient with altered mental status in an oral boards format; 2) review appropriate laboratory testing and diagnostic imaging; 3) identify signs and symptoms of thyroid storm and 4) review appropriate pharmacologic therapies with the proper sequence and timing.

**Educational Methods:**

Practice boards case.

**Research Methods:**

This oral boards practice case was developed and then tested in several small group settings. First, EM resident learners discussed the case in a small group format. Their feedback was utilized to refine the case’s textual information. Subsequently, EM physicians preparing for the ABEM oral board examination provided additional general feedback of the case and completed an anonymous survey regarding case quality and educational value.

**Results:**

Minor changes were made based on feedback from small group sessions. The finalized case was tested with individuals, and surveys showed that 92% (12/13) of individuals rated the case quality as excellent (standard Likert scale 1–5 with 5 being Excellent). All participants responded affirmatively that the case enhanced their understanding of thyroid storm.

**Discussion:**

This oral boards practice case was effective in preparing learners for the ABEM oral boards exam. Based on learner feedback, several laboratory results were added to the stimulus package and wording was edited to improve the clarity of the case.

**Topics:**

Hyperthyroidism, thyrotoxicosis, thyroid storm, endocrine emergencies, altered mental status.

## USER GUIDE

**Table:** 

List of Resources:	
Abstract	1
User Guide	3
For Examiner Only	5
Oral Boards Assessment	11
Stimulus	15
Debriefing and Evaluation Pearls	30

**Learner Audience:**

Medical students, interns, junior residents, senior residents

**Time Required for Implementation:**

Case: 10 minutes as a single case

Debriefing: 15 minutes

**Learners per instructor:**

This case can be practiced with one learner per instructor or a small group observational setting.

**Topics:**

Hyperthyroidism, thyrotoxicosis, thyroid storm, endocrine emergencies, altered mental status.

**Objectives:**

By the end of this oral boards case, learners will be able to:

1. Assess a patient with altered mental status in an oral boards format.
2. Review appropriate laboratory testing and diagnostic imaging for a patient with altered mental status.
3. Identify signs and symptoms of thyroid storm.
4. Review appropriate pharmacologic therapies with the proper sequence and timing.

### Linked objectives and methods

An oral boards format was selected as an alternative modality to teach this core EM topic in order to provide real-time feedback to the learner as the case progresses. Symptoms of thyroid storm are often initially non-specific and may mimic many other clinical entities, making this teaching style beneficial because learners must form a large differential diagnosis and remember to suspect endocrine abnormalities as a possible cause (Objective 1). After initial evaluation, learners must then order, review, and interpret appropriate laboratory testing and diagnostic imaging with attention given to information which leads them to the ultimate diagnosis (Objective 2). Based on the information provided, learners should hone in on signs and symptoms which point to thyroid storm (Objective 3). After establishing thyroid storm as the appropriate diagnosis, learners should order appropriate pharmacologic interventions in the proper order for treatment (Objective 4). The use of a one-on-one practice case format enables the teacher to tailor additional information to various levels of training when providing further information on clinical pearls after the case conclusion. Any gaps in knowledge may be identified by the examiner and then addressed in a focused debrief or mini-lecture after the conclusion of the practice case.

### Recommended pre-reading for instructor

- Awad N. Thyroid storm: treatment strategies. Academic Life in Emergency Medicine. https://www.aliem.com/2013/11/thyroid-stormtreatment-strategies/. Published November 11, 2013. Accessed on February 7, 2019.
- Idrose A. Hyperthyroidism and Thyroid Storm. In: Tintinalli JE, Ma OJ, Yealy DM, et al eds. *Tintinalli’s Emergency Medicine: A Comprehensive Study Guide*. 9^th^ ed. New York, NY: McGraw-Hill; 2019; 1450–1456.
- Field A. Thyroid storm. In: Wolfson AB, ed. *Harwood-Nuss’ Clinical Practice of Emergency Medicine*. 6^th^ ed. Philadelphia, PA: Lippincott Williams & Wilkins; 2014; 1025–1028.

### Results and tips for successful implementation

This case was designed to be used as preparation for the emergency medicine oral board examination. Faculty members may administer the case to residents by presenting the initial case information and then requesting for residents to verbalize their evaluation and clinical treatment plan. In addition, it may be used as part of a didactic teaching curriculum, which begins with the case presentation and then focuses on key teaching points as a mini-lecture following the case resolution.

This oral boards case was developed and then previewed in small group settings to ensure inclusive information and to resolve any ambiguities of the case. The finalized version was then tested with thirteen physicians who were preparing for the ABEM oral boards and a survey was administered to assess case quality and educational value. 92% (12/13) of these physicians rated the case as excellent (standard Likert scale 1–5 with 5 being Excellent), and all residents responded affirmatively that the case enhanced their understanding of thyroid storm. Learners noted that the case was “complex” and “allowed for higher order thinking,” also stating that “there were subtle features that should clue the examinee not to anchor on sepsis” and that “it was helpful to think through the differential for a hyperthermic altered patient.” Based on feedback from these sessions, additional learning points were clarified.

## Figures and Tables

**Table:** 

**Vitals:** HR 142	BP 158/99	RR 24	Temp 40.1°C	O_2_Sat 98% on room air

**Table:** 

	Milestone	Did not achieve level 1	Level 1	Level 2	Level 3
**1**	**Emergency Stabilization (PC1)**	□ **Did not achieve Level 1**	□ **Recognizes abnormal vital signs**	□ **Recognizes an unstable patient, requiring intervention** **Performs primary assessment** **Discerns data to formulate a diagnostic impression/plan**	□ **Manages and prioritizes critical actions in a critically ill patient** **Reassesses after implementing a stabilizing intervention**
**2**	**Performance of focused history and physical (PC2)**	□ **Did not achieve Level 1**	□ **Performs a reliable, comprehensive history and physical exam**	□ **Performs and communicates a focused history and physical exam based on chief complaint and urgent issues**	□ **Prioritizes essential components of history and physical exam given dynamic circumstances**
**3**	**Diagnostic studies (PC3)**	□ **Did not achieve Level 1**	□ **Determines the necessity of diagnostic studies**	□ **Orders appropriate diagnostic studies** **Performs appropriate bedside diagnostic studies/procedures**	□ **Prioritizes essential testing** **Interprets results of diagnostic studies** **Considers risks, benefits, contraindications, and alternatives to a diagnostic study or procedure**
**4**	**Diagnosis (PC4)**	□ **Did not achieve Level 1**	□ **Considers a list of potential diagnoses**	□ **Considers an appropriate list of potential diagnosis** **May or may not make correct diagnosis**	□ **Makes the appropriate diagnosis** **Considers other potential diagnoses, avoiding premature closure**
**5**	**Pharmacotherapy (PC5)**	□ **Did not achieve Level 1**	□ **Asks patient for drug allergies**	□ **Selects an appropriate medication for therapeutic intervention, considering potential adverse effects**	□ **Selects the most appropriate medication(s) and understands mechanism of action, effect, and potential side effects** **Considers and recognizes drug-drug interactions**
**6**	**Observation and reassessment (PC6)**	□ **Did not achieve Level 1**	□ **Reevaluates patient at least one time during the case**	□ **Reevaluates patient after most therapeutic interventions**	□ **Consistently evaluates the effectiveness of therapies at appropriate intervals**
**7**	**Disposition (PC7)**	□ **Did not achieve Level 1**	□ **Appropriately selects whether to admit or discharge the patient**	□ **Appropriately selects whether to admit or discharge** **Involves the expertise of some of the appropriate specialists**	□ **Educates the patient appropriately about their disposition** **Assigns patient to an appropriate level of care (ICU/Tele/Floor)** **Involves expertise of all appropriate specialists**
**22**	**Patient centered communication (ICS1)**	□ **Did not achieve level 1**	□ **Establishes rapport and demonstrates empathy to patient (and family)** **Listens effectively**	□ **Elicits patient’s reason for seeking health care**	□ **Manages patient expectations in a manner that minimizes potential for stress, conflict, and misunderstanding**.
**23**	**Team management (ICS2)**	□ **Did not achieve level 1**	□ **Recognizes other members of the patient care team during case (nurse, techs)**	□ **Communicates pertinent information to other healthcare colleagues**	□ **Communicates a clear, succinct, and appropriate handoff with specialists and other colleagues** **Communicates effectively with ancillary staff**

**Table:** 

**#1**	**Patient information form**
**#2**	**Complete blood count (CBC)**
**#3**	**Comprehensive metabolic panel (CMP)**
**#4**	**Urinalysis**
**#5**	**Pregnancy Test**
**#6**	**Urine Drug Screen**
**#7**	**Serum Toxicology Screen**
**#8**	**Troponin**
**#9**	**Creatine Kinase**
**#10**	**Thyroid Function Studies**
**#11**	**Lactate**
**#12**	**Arterial blood gas**
**#13**	**Electrocardiogram (ECG)**
**#14**	**Chest radiograph**
**#15**	**Head Computed Tomography (CT)**

**Table:** 

**Patient’s Name:**		**Andrea Mason**
**Age:**		**33**
**Gender:**		**Female**
**Chief Complaint:**		**Altered mental status**
**Person Providing History: EMS and patient’s husband**		
**Vital Signs:**		
	**Temp:**	**40.1°C**
	**BP:**	**158/199**
	**P:**	**142**
	**RR:**	**24**
	**O** **_2_** **sat:**	**98% (room-air)**
	**Weight:**	**65 kg**

**Table:** 

**White blood cell count (WBC)**	**16.1 x1000/mm3**
**Hemoglobin (Hgb)**	**12.9 g/dL**
**Hematocrit (Hct)**	**38.8%**
**Platelets**	**270 x1000/mm3**
**Neutrophils**	**66% (10,626)**
**Lymphocytes**	**26% (4,186)**
**Monocytes**	**5% (805)**
**Eosinophils**	**2.5% (402)**
**Basophils**	**0.5% (81)**

**Table:** 

**Sodium**	**142 mEq/L**
**Potassium**	**4.4 mEq/L**
**Chloride**	**107 mEq/L**
**Bicarbonate**	**24 mEq/L**
**Blood Urea Nitrogen (BUN)**	**13 mg/dL**
**Glucose**	**95 mg/dL**
**Creatinine (Cr)**	**0.68 mg/dL**
**Calcium**	**9.4 mg/dL**
**Anion Gap**	**11**
**Direct Bilirubin**	**0.2 mg/dL**
**Total Bilirubin**	**0.4 mg/dL**
**Albumin**	**4.1 g/dL**
**Alkaline Phosphatase**	**49 Units/L**
**ALT**	**11 Units/L**
**AST**	**15 Units/L**
**Total Protein**	**7.3 g/dL**

**Table:** 

**Color**	**Yellow**
**Appearance**	**Cloudy**
**Sp Gravity**	**1.023**
**pH**	**5.5**
**Glucose**	**Negative**
**Protein**	**30**
**Ketones**	**Negative**
**Bilirubin**	**Negative**
**Leukocyte Esterase**	**Large**
**Nitrite**	**Positive**
**Blood**	**Small**
** ** **Microscopy**	
** ** **WBC**	** ** **1123/high powered field (hpf)**
** ** **Red blood cells (RBC)**	** ** **35/hpf**
** ** **Bacteria**	** ** **3+**

**Table:** 

**Acetaminophen**	**Negative**
**Amphetamine**	**Negative**
**Barbituate**	**Negative**
**Benzodiazepine**	**Negative**
**Buprenorphine**	**Negative**
**Cannabinoid**	**Negative**
**Cocaine**	**Negative**
**Ethanol**	**Negative**
**Methadone**	**Negative**
**Opiate**	**Negative**
**Oxycodone**	**Negative**
**Salicylate**	**Negative**
**Tricyclic**	**Negative**

**Table:** 

**Acetaminophen**	**Negative**
**Ethanol**	**Negative**
**Salicylate**	**Negative**

**Table:** 

**Troponin**	**<0.01 ng/mL**

**Table:** 

**CK**	**380 Units/L**

**Table:** 

**TSH**	**0.04 mIU/L (normal 0.350 – 3.600)**
**Free T4**	**17.4 ng/dL (normal 0.70 – 1.37)**

**Table:** 

**Lactate**	**2.4 mEq/L**

**Table:** 

**pH**	**7.41**
**pCO2**	**37 mmHg**
**pO2**	**94 mmHg**
**HCO3**	**26 mmol/L**

**Figure:**
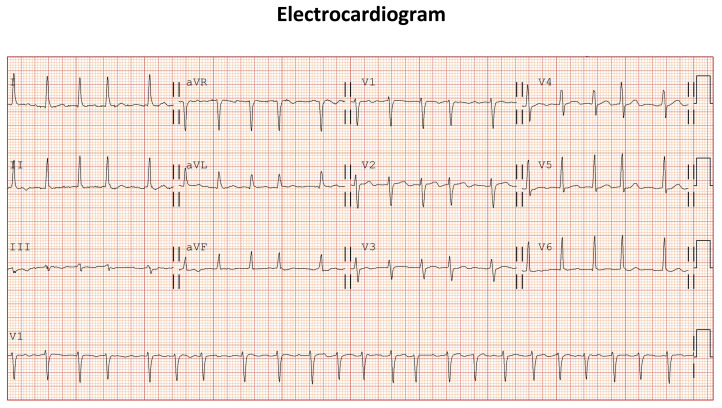
Image Citation: Author’s own image

**Figure:**
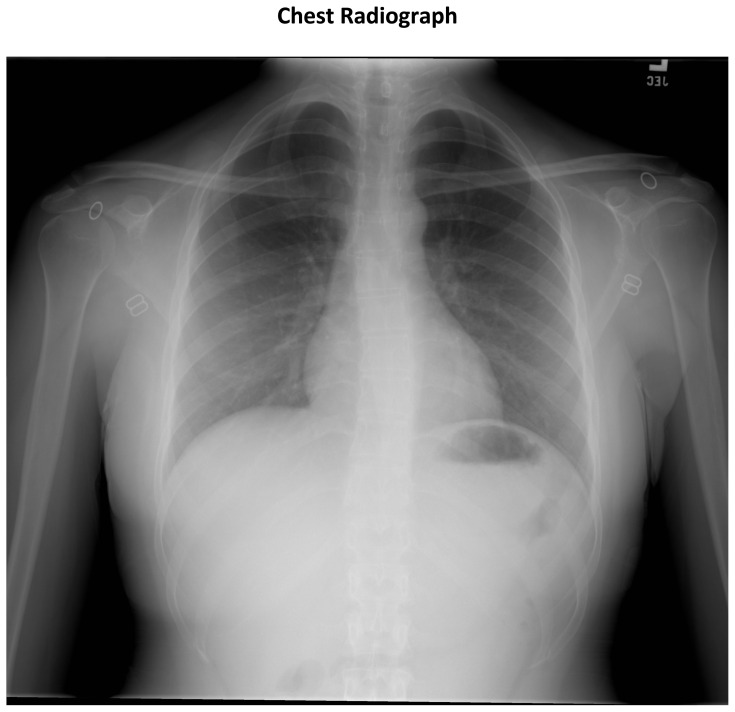
Image Citation: Author’s own image

**Figure:**
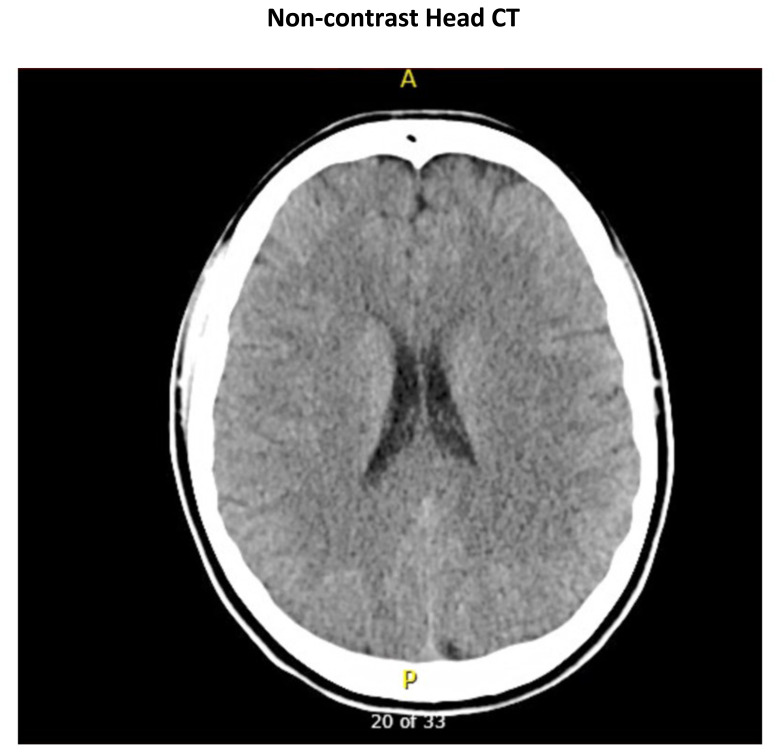
Image Citation: Author’s own image

**Table:** Treatment of Thyroid Storm

**Block peripheral thyroid hormone effects**	Propranolol, esmolol
**Inhibit release of thyroid hormones**	Propylthiouracil, methimazole
**Decrease peripheral conversion of T4 to T3**	Hydrocortisone, dexamethasone
**Inhibit production of new thyroid hormones**	Lugol solution, potassium iodide

## FOR EXAMINER ONLY

### Oral Case Summary

**Diagnosis: Thyroid Storm**

**Case Summary:** A 33-year-old female with a past medical history of celiac disease presents to the emergency department via emergency medical services with altered mental status. The patient’s husband is at the bedside and provides further history. Her physical exam is remarkable for altered mental status, diaphoresis, palpable goiter, and an irregularly irregular cardiac rhythm.

**Order of Case:** The learner should start by assessing the patient’s general appearance and ABCs. Vital signs should be obtained. With the patient’s altered mental status, a point-of-care glucose should be ordered early in the case. A full history and physical exam should be performed. Labs and computed tomography (CT) of the head should be ordered. Sepsis will be on the differential, so treatment with broad-spectrum antibiotics should be initiated. The patient’s clinical presentation will be consistent with thyroid storm, and the learner should start treatment. It is important that the learner specifies that they will not administer any iodine-containing products until at least one hour after the thionamides (propylthiouracil or methimazole). The learner should update the family about the patient’s plan of care, and this patient should be admitted to the medical intensive care unit.

**Disposition:** Admit to the intensive care unit.

**Critical Actions:**

1. Perform a complete history and physical exam.
2. Obtain a point-of-care blood glucose.
3. Order appropriate labs and imaging: complete blood count (CBC), comprehensive metabolic panel (CMP), urinalysis with culture, pregnancy test, urine toxicologic screen, acetaminophen level, ethanol level, salicylate level, troponin, creatine kinase (CK), thyroid function studies, lactic acid, arterial blood gas (ABG), blood cultures, electrocardiogram (ECG), chest X-ray, and CT head.
4. Begin broad-spectrum antibiotics.
5. Start medications for treatment of thyroid storm with the proper sequence and timing.
6. Admit the patient to the intensive care unit.

### Historical Information

**Chief Complaint:** Confusion

**History of present illness:** A 33-year-old female presents to the emergency department via EMS with altered mental status. The patient is confused and cannot provide further history, but the patient’s husband states that she had been complaining of some burning pain with urination for the past 3 days. She had been taking over-the-counter cranberry supplementation at home for these symptoms. The patient’s husband went home to check on his wife after her coworkers called him stating that she did not show up to work that morning. He found her diaphoretic, laying on the couch. She was confused and could only tell her husband her name. He called EMS, who transported the patient to the emergency department for evaluation and treatment.

**Past Medical history:** Celiac disease

**Past Surgical history:** Cesarean section three years ago

**Patient’s Medications:** Daily multivitamin, combined oral contraceptive

**Allergies:** No known drug allergies

**Social history:**

- Tobacco: Denies
- Alcohol: Drinks one glass of wine two nights per week
- Drug use: Denies

**Family history:** No known medical problems run in the family

### Physical Exam Information

**Weight:** 65 kg

**General appearance:** Laying on the stretcher, diaphoretic.

**Primary survey:**

- **Airway:** Patent and protected.
- **Breathing:** Tachypneic. Breath sounds clear and equal bilaterally without any wheezing, crackles, or rhonchi.
- **Circulation:** 2+ distal pulses.

**Physical examination:**

- **General appearance:** Laying on the stretcher, diaphoretic.
- **Head, eyes, ears, nose and throat (HEENT):**
  ○ **Head:** Normocephalic, atraumatic.
  ○ **Eyes:** Pupils are 3 mm bilaterally and reactive to light. Normal conjunctivae. Proptosis bilaterally. Lid lag with extraocular movements.
  ○ **Ears:** External ears normal bilaterally. Normal tympanic membranes bilaterally.
  ○ **Nose:** Normal.
  ○ **Throat/Oropharynx:** Dry mucous membranes. Oropharynx clear without any erythema or exudates.
- **Neck:** Supple, full range of motion. No meningismus. Palpable goiter.
- **Cardiovascular:** Irregularly irregular rhythm, rapid rate, no murmurs.
- **Respiratory:** Tachypneic. Breath sounds clear and equal bilaterally without any wheezing, crackles, or rhonchi.
- **Abdomen:** Soft, nondistended. Suprapubic tenderness to palpation. No rebound tenderness or involuntary guarding.
- **GYN:** No vaginal discharge or bleeding. Normal, closed cervix. No cervical motion tenderness. No adnexal tenderness or masses.
- **Rectal:** Normal rectal tone. No gross blood. Guaiac negative stool.
- **Back:** No midline tenderness to palpation. Right costovertebral angle tenderness.
- **Extremities:** Normal range of motion of joints with no rigidity.
- **Neuro:** Agitated. Oriented to self only. Opens eyes to voice. Follows commands in all 4 extremities. Cranial nerves II–XII intact. 5/5 strength and intact sensation to light touch in all 4 extremities. No clonus. Hyperreflexia.
- **Skin:** Diaphoretic. No rashes.
- **Lymph:** No lymphadenopathy.
- **Psych:** Unable to completely assess since patient is only able to state her name.

### Critical Actions and Cueing Guidelines

1. **Perform a complete history and physical exam**.
  The learner should perform a complete history and physical exam, including a neurologic exam in the setting of altered mental status. When the patient cannot completely answer questions, the learner should obtain the history from the patient’s husband at the bedside.
  - Cueing Guideline (if applicable):
    If the learner has not completed a history and physical exam, the examiner may cue the learner by the husband asking, “Doctor, why is my wife so confused?”
2. **Obtain a point-of-care blood glucose**.
  The learner should ask for a point-of-care blood glucose early in the evaluation of the patient before ordering additional labs and imaging.
  - Cueing Guideline (if applicable):
    If the learner has not yet ordered a point-of-care blood glucose, the examiner may cue the learner by the nurse stating, “It might take some time to get an IV in this patient, so is there anything we can check quickly at the bedside to determine the cause of this patient’s condition?”
3. **Order appropriate labs and imaging: CBC, CMP, urinalysis with culture, pregnancy test, urine toxicologic screen, acetaminophen level, ethanol level, salicylate level, troponin, CK, thyroid function studies, lactate, ABG, blood cultures, ECG, chest X-ray, and CT head**.
  The learner should order appropriate labs and imaging as part of the diagnostic evaluation of a patient with fever and altered mental status.
  - Cueing Guideline (if applicable):
    If the learner has not yet ordered any diagnostic studies, the examiner may cue the learner by the nurse asking, “Doctor, do you want to place any orders?”
4. **Begin broad-spectrum antibiotics**.
  With the patient’s fever and altered mental status, the learner should empirically start broad-spectrum antibiotics.
  - Cueing Guideline (if applicable):
    If the learner has not ordered any antibiotics, the examiner may cue the learner by the nurse stating, “Do we need to do anything to treat this patient’s tachycardia and fever?”
5. **Start medications for treatment of thyroid storm in the correct order**.
  The learner should start the patient on a beta-blocker, thionamide, glucocorticoid, and iodine therapy. However, the iodine therapy should not be started until at least 1 hour after the first dose of thionamide.
  - Cueing Guideline (if applicable):
    If the learner has not ordered any therapy yet after receiving the lab work, the nurse should ask, “The pharmacy called and was wondering if there are any medications that they should start preparing for this patient with all those critical alerts from the lab.”
6. **Admit the patient to the intensive care unit**.
  After recognizing that this patient has thyroid storm and initiating appropriate therapies for this condition, the learner should determine that this patient will require admission to the intensive care unit.
  - Cueing Guideline (if applicable):
    If the learner has not yet admitted this patient, the examiner may cue the learner by the husband asking, “Is my wife going to be able to come home tonight?”

## ORAL BOARDS ASSESSMENT

*Thyroid Storm*

Learner: _________________________________________

**Critical Actions:**

□ Perform a complete history and physical exam
□ Obtain a point-of-care blood glucose
□ Order appropriate labs and imaging: CBC, CMP, urinalysis with culture, pregnancy test, thyroid function studies, blood cultures, EKG, chest x-ray, and CT head
□ Begin broad-spectrum antibiotics
□ Start medications for treatment of thyroid storm in the correct order
□ Admit the patient to the intensive care unit

**Summative and formative comments:**

**Milestone assessment:**

## Stimulus #5

### Pregnancy Test

**Negative**

## DEBRIEFING AND EVALUATION PEARLS

### Thyroid Storm

- Thyroid storm occurs in 1–2% of patients with hyperthyroidism and may be precipitated by infection, surgery, pregnancy, trauma, thyroid medication changes, or iodinated contrast exposure.
- Thyroid storm is a clinical diagnosis. The Burch-Wartofsky point scale can be used to determine when thyrotoxicosis progresses to thyroid storm.
- Therapy is targeted at blocking peripheral thyroid hormone effects, inhibiting the release of thyroid hormones, decreasing peripheral conversion of T4 to T3, and inhibiting production of new thyroid hormones.
- Propylthiouracil should be used in the first trimester of pregnancy due to teratogenicity of methimazole at that time. However, methimazole should be used in the second and third trimesters.
- Pharmacologic therapies to inhibit the production of new thyroid hormones should be given at least one hour after thionamides (propylthiouracil or methimazole) to prevent stimulation of new thyroid hormone synthesis.
- Steroids have the additional benefit of treating adrenal insufficiency in addition to decreasing the peripheral conversion of T4 to T3.
- Infection is a common trigger of thyroid storm, and these patients should be treated with broad-spectrum antibiotics.
- Patients with thyroid storm will require admission to the intensive care unit.
